# Utility of routine exercise treadmill testing early after percutaneous coronary intervention

**DOI:** 10.1186/1471-2261-7-12

**Published:** 2007-03-29

**Authors:** Mohan N Babapulle, Jean G Diodati, James C Blankenship, Thao Huynh, Sabrina Cugno, Radha Puri, Phuong A Nguyen, Mark J Eisenberg

**Affiliations:** 1 Division of Cardiology, St. Mary's Regional Cardiac Care Centre Kitchener, Ontario, Canada; 2 Division of Cardiology, Sacre Coeur Hospital, Montreal, Quebec, Canada; 3 Department of Cardiology, Geisinger Medical Center, Danville, Pennsylvania, USA; 4 Division of Cardiology, Montreal General Hospital, Montreal, Quebec, Canada; 5 Divisions of Cardiology and Clinical Epidemiology, Jewish General Hospital, Montreal, Quebec, Canada

## Abstract

**Background:**

There have been few prospective studies examining the utility of routine exercise treadmill testing (ETT) early after percutaneous coronary intervention (PCI). The objective of this study was to examine the impact of a routine ETT strategy early after PCI on follow-up cardiac events and procedures.

**Methods:**

We examined 136 patients who underwent routine ETT at 6 weeks post-PCI in the ADORE trial. The ETT was classified as positive, indeterminate, or negative. The Duke Treadmill Score (DTS) was calculated for all patients. Follow-up occurred at 9 months.

**Results:**

ETT results at 6 weeks were: 32 (23.5%) positive, 24 (17.6%) indeterminate and 80 (58.8%) negative. At 9 months, the composite event rate was 21.9% in those with a positive ETT, 20.8% in those with an indeterminate ETT and 12.5% in those with a negative ETT (p = 0.25 positive vs. negative ETT). The sensitivity of early ETT for predicting clinical events was 41.2%, the specificity was 73.3%, the positive predictive value was 21.9% and the negative predictive value was 87.5%. At 9 months, the cardiac procedure rate was 18.8% in those with a positive test, 13.0% in those with an indeterminate test, and 6.3% in those with a negative test (p = 0.07 positive vs. negative ETT). In a multivariate logistic regression model, coronary stenting during PCI and a ≥ 85% MPHR achieved were found to be inversely associated with clinical events. However, the DTS did not independently predict clinical events.

**Conclusion:**

Although the statistical power of the study was limited by the small number of clinical events (particularly MI and death), the results of this study support the ACC/AHA guidelines that exercise treadmill testing should not be used routinely after PCI.

## Background

Over one million percutaneous coronary interventions (PCI) are performed worldwide annually. The rate of restenosis is between 30–40% following balloon angioplasty and 20–30% following stenting with bare metal stents, and <10 % with drug-eluting stents [1-4]. Most patients with restenosis present with stable or unstable angina, although a significant proportion of patients with restenosis are asymptomatic [5-7]. Some physicians routinely perform exercise treadmill testing (ETT) early after PCI in order to identify those patients with residual stenoses, to assess functional capacity, or to identify silent ischemia [8,9]. However, it is unclear if a routine early ETT strategy is predictive of clinical events following PCI. Two studies examining the role of ETT post-PCI suggest that it may predict events (restenosis) [10,11], although the majority of observational studies suggest that it does not [12-16]. The current ACC/AHA guidelines for exercise stress testing suggest that routine screening with ETT early after PCI is not indicated. However, exercise stress testing may be of value in high risk patients [17]. Our hypothesis was that a routine, 6-week ETT is not predictive of clinical events.

## Methods

The Aggressive Diagnosis of Restenosis (ADORE) trial was a prospective, multicenter trial that randomized patients post-PCI to a strategy of routine functional testing vs. a selective, symptom-driven functional testing strategy. Prior to patient enrollment, ethics approval was obtained from the institutional review boards of all participating centers. Only patients who were completely revascularized were enrolled. Patients in the routine arm of the trial underwent an ETT 6 weeks following the index PCI. The ETT's were performed while the patients were taking their usual medications, including antianginals. The ETT's were interpreted by the physician supervising the test, and the results forwarded to the patients' treating physicians. Clinical follow-up occurred at 9 months following randomization. Data on clinical events (unstable angina, myocardial infarction, death,) and repeat cardiac procedures (coronary angiography, repeat PCI, coronary artery bypass grafting) during the study period were obtained.

We conducted a post-hoc analysis of the subgroup of patients that underwent early ETT in the routine arm of the trial. Of the 176 patients that were randomized to routine 6-week ETT, 146 (83%) underwent an ETT between 5 and 7 weeks after index PCI, and 136 (93%) had complete follow-up data available. These 136 patients with complete follow-up data formed the study cohort. The exercise ECG was regarded as being adequate for diagnosis of cardiac ischemia if the baseline ECG at rest demonstrated ST depression < 1 mm, and there was no evidence of left ventricular hypertrophy or a left bundle branch block pattern [17]. The ETT was performed and interpreted according to standard criteria and definitions [17,18]. A clinically positive ETT was defined as the development of angina or angina-equivalent with exercise. An electrically positive ETT was defined as ≥ 1 mm ST-segment depression in ≥ 3 consecutive beats persisting ≥ 1 minute into recovery. The ETT results were classified as positive (clinically or electrically positive), negative (clinically and electrically negative), or indeterminate (inability to achieve ≥ 85% MPHR with no ECG changes). In the 3 study groups, we analyzed clinical outcomes (unstable angina, myocardial infarction, death) and rates of cardiac procedures (coronary angiography, repeat PCI, and coronary artery bypass graft surgery). We also calculated the Duke Treadmill Score (DTS) for all patients [19,20]. This score is calculated by the following formula: DTS = exercise time on a standard Bruce protocol in minutes - 5 × (ST-segment depression in mm) - 4 × (angina score). The angina score is 0 for no exercise-induced angina, 1 for non-exercise limiting angina, and 2 for exercise-limiting angina. If a treadmill protocol other than the standard Bruce were used, the exercise time corresponding to the standard Bruce was calculated as follows: exercise time = (METS +2.2)/1.3 [21]. A lower DTS (especially ≤ -10) indicates more severe ischemia and higher mortality than a higher score.

### Data analysis

Continuous data are presented as the mean ± standard deviation, and dichotomous data are presented as percentages. Descriptive analyses examined the baseline clinical and procedural characteristics of patients undergoing PCI. Statistical analyses used the Fisher's exact test for dichotomous variables. Using logistic regression, we also examined baseline characteristics, procedural characteristics, and functional testing results in order to determine correlates of clinical outcomes during the 9 months following PCI.

## Results

### Baseline characteristics

Baseline clinical and procedural characteristics are outlined in Table 1. In general, the study patients were middle-aged men who underwent PCI with stent implantation for single-vessel coronary artery disease. Procedural success was high, and low residual stenosis rates were observed. Differences in baseline clinical and procedural characteristics were observed between the 3 groups of patients. Those in the positive ETT group were older, had a higher proportion of women, and were more likely to have multi-vessel coronary artery disease than those with an indeterminate or negative ETT. Patients with a positive ETT were also less likely to be diabetic.

### Six-week exercise treadmill test

Most patients had the ETT while on antianginal medications, including beta-blockers. The baseline ECG was normal in most patients, and was adequate for the diagnosis of ischemia in >80% of the patients. Among the 136 patients, 32 (23.5%) patients had a positive ETT (development of exertional angina in 56.3% and reversible ischemia as noted by ECG changes in 87.5% of patients), 24 (17.6%) had an indeterminate ETT, and 80 (58.8%) had a negative ETT. A number of patients in each of the 3 groups did not reach ≥ 85% of their maximal age-predicted heart rate (MPHR, defined as 220-age). However, most patients achieved a good cardiac workload, reaching a MET level ≥ 6.0 and a Rate-Pressure Product ≥ 20,000. The Duke Treadmill Score was lower in those patients with positive and indeterminate ETT's, compared with those with a negative ETT [Table 2].

### Clinical events and cardiac procedures

The rates of clinical events and cardiac procedures are listed in Table 3. The majority of events were due to unstable angina. There was no difference in the composite event rate between the groups with a positive vs. negative ETT. There was a trend toward a higher rate of repeat cardiac procedures (coronary angiography, repeat PCI, or CABG) after the index PCI in the group with a positive ETT compared with those with a negative ETT, with a two-fold difference in procedure rates (p = 0.07 positive vs. negative ETT). There was no statistically significant difference in event rates between the positive and indeterminate ETT groups combined and the negative ETT group.

### Test characteristics of a 6-week ETT

Excluding those patients with an indeterminate ETT, the sensitivity of a routine early ETT at predicting clinical events was 41.2%, the specificity was 73.7%, the positive predictive value was 21.9% and the negative predictive value was 87.5%. If the positive and indeterminate ETT groups were combined, the sensitivity of the ETT improved to 54.5%, but specificity decreased to 61.4%. The positive predictive value decreased slightly to 21.6%, and there was no change in the negative predictive value.

### Univariate and multivariate regression analyses

By univariate analysis, several variables were correlated with the occurrence of clinical events [Table 4]. However, when these variables were tested in a multivariate logistic regression model, only coronary stenting during PCI and a ≥ 85% MPHR achieved were found to be inversely associated with clinical events. The maximum cardiac workload (MET level) achieved, the Rate-Pressure product, and the Duke Treadmill Score did not correlate with clinical events in our regression model.

## Discussion

The objective of our study was to examine the impact of routine early ETT on follow-up cardiac events and procedures. We hypothesized that a routine, 6-week ETT following PCI is not predictive of clinical events, and that a positive ETT would result in a higher rate of repeat cardiac procedures. We found that an early ETT was poorly predictive of clinical events, with a positive predictive value of only 21.9%. The composite endpoint of clinical events included diagnoses of unstable angina, MI, or death. Because the diagnosis of unstable angina does not necessarily correlate with long term consequences, we considered this diagnosis to be a soft endpoint. Since the composite endpoint of clinical events was largely driven by the diagnosis of unstable angina, we believe that early ETT is a poorer predictor of hard endpoints of MI and death. We also found that a positive ETT result may be associated with a higher rate of repeat cardiac procedures. In contrast, the high negative predictive value for ETT observed in our analysis (87.5%) reflects the low rate of clinical events in our study group. These results suggest that an ETT early after PCI is of little clinical utility, and they support the current guidelines of the ACC/AHA on functional testing after PCI [17]. However, definitive conclusions cannot be drawn from our study due to the relatively small numbers of patients and clinical events.

In an effort to identify patients with significant residual stenoses and to assess functional capacity post-revascularization, some physicians perform ETT early (< 2 months) after PCI [8,9,15]. The results of a few observational studies examining the utility of an ETT in asymptomatic patients do not support the practice of routine ETT following PCI [12-16]. The current ACC/AHA guidelines for exercise stress testing post-PCI do not advocate routine screening ETT for the detection of restenosis in all patients. However, they conclude that there are insufficient data to support either a routine or a selective ETT strategy, especially in patients at a high risk of restenosis [17].

The results of our study are in keeping with other studies investigating the utility of an early ETT at predicting angiographic restenosis [23-25]. In these studies, the sensitivity of the ETT at predicting restenosis ranged between 59–83% and the specificity between 32–66%. The positive predictive value was also poor (44–59%). However, these were observational studies and they did not investigate clinical end points. In contrast with other studies examining this issue, our study examined clinical event rates in patients from a cohort randomized to undergo a routine early ETT as part of the ADORE trial, and the impact of such an ETT on follow-up cardiac procedures, thereby limiting a possible selection bias.

The poor sensitivity of an early ETT at predicting restenosis and clinical events is due to several factors. Given that a majority of patients in our study underwent coronary stenting, it is unlikely that a significant degree of restenosis will have occurred prior to 6 weeks following PCI; thereby making ETT an unessential exam in the follow-up period of post-stent implantation. ETT is also thought to have a lower sensitivity for the detection of ischemia involving the territory of a single coronary vessel, compared with multi-vessel CAD [21]. Angina post-PCI is also an insensitive marker of restenosis as up to 50% of patients have cardiac ischemia that is clinically silent [5-7]. An early ETT also has a low specificity at identifying patients at a higher risk of restenosis primarily due to ST-segment shifts with exercise unrelated to ischemia. It has been demonstrated that ST-segment changes with exercise may persist following successful PCI, and is not always correlated with restenosis [14]. Spasm of small coronary vessels may be responsible for these electrically positive tests in the absence of angiographic restenosis, although the exact mechanism has yet to be elucidated [26].

In order to increase the diagnostic accuracy of the ETT, several authors have published novel predictors of restenosis. An increase in the ΔST/ΔHR index on serial ETT's appears to significantly improve the diagnostic accuracy of an ETT following balloon angioplasty for single vessel coronary artery disease [23]. Other possible predictors of restenosis of an early ETT have also been identified [24,25,27]. However, the diagnostic accuracy for restenosis of these novel predictors has not been extensively investigated, and their calculation remains technically difficult.

Our study also raises important questions about the utility of an early ETT following PCI. By multivariate logistic regression modeling we demonstrated an association between <85% MPHR achieved with composite clinical events in our study. Our analysis shows that inability to achieve ≥ 85% of the MPHR on an ETT after complete coronary revascularization is an independent predictor of clinical events. To our knowledge, this is the first report of prognostic utility of a 6-week ETT following PCI for stable CAD. We feel that ability to achieve this target heart rate is a surrogate marker of exercise capacity as measured by the MET level achieved. This finding is in general agreement with other studies that have shown independent prognostic value of exercise capacity (as measured by the MET level achieved and the RPP) in other groups of patients [28-32]. Our finding is also in agreement with a recent study demonstrating a two-fold higher mortality rate in asymptomatic women who failed to achieve ≥ 85% of their age-predicted MPHR [33]. However, the maximum MET level achieved and the Rate-Pressure Product did not correlate with clinical events in our study. This may have been due to the low event rate observed and a low proportion of patients with multi-vessel CAD in our study. An early ETT following PCI may help physicians identify this subgroup of patients with a poor exercise capacity with a higher risk of future coronary events. Early ETT may also be of value to assess a patient's exercise capacity with regard to rehabilitation and their return to the workforce. These patients, similar to other patients with obstructive CAD, may benefit from aggressive modification of risk factors for CAD with behavioral changes and medical therapy.

## Limitations

There are several limitations to this study. First, the relatively small number of patients and clinical events limits the statistical power of our analysis, and increases the likelihood of a type II (beta) error. Second, the lack of a standardized protocol for performing the ETT in our study may have lead to inter-operator variability. However, our intention was to provide a snapshot of real world practice within the setting of a clinical trial. Third, the continuation of beta blockers could lower maximum heart rate, thus confusing this variable. Finally, antianginal therapy was not stopped routinely prior to ETT, which may have delayed the time to the onset of cardiac ischemia. Since all patients enrolled in the ADORE trial were completely revascularized, and most patients achieved an excellent cardiac workload, this is unlikely to have had a significant impact on the ETT results.

## Conclusion

Our study was designed to examine differences in clinical outcome and repeat cardiac procedure rates in patients undergoing routine ETT 6 weeks after PCI. We found that there did not appear to be a significant difference in clinical events between those with a positive ETT compared with those with a negative ETT, although definitive conclusions cannot not be drawn due to limited statistical power. An early ETT also appears to be poorly predictive of clinical events and, a positive ETT result may lead to a higher rate of repeat cardiac procedures. These results suggest that a routine early ETT following PCI has little clinical utility, and support the current consensus guidelines of the ACC/AHA that routine exercise treadmill testing early after PCI is not predictive of subsequent clinical events.

## Competing interests

The author(s) declare that they have no competing interests.

## Authors' contributions

MB drafted the manuscript, and read and approved the final manuscript, JD enrolled patients, contributed to the manuscript, and read and approved the final manuscript, JB enrolled patients, contributed to the manuscript, and read and approved the final manuscript, TH enrolled patients, contributed to the manuscript, and read and approved the final manuscript, PN did data entry and read and approved the final manuscript, SC performed the analysis, contributed to the manuscript, and read and approved the final manuscript, RP performed the analysis, contributed to the manuscript, and read and approved the final manuscript, ME performed the study, enrolled patients, supervised the analysis, contributed to the manuscript, and read and approved the final manuscript.

## Pre-publication history

The pre-publication history for this paper can be accessed here:



## Figures and Tables

**Table 1:** Baseline Clinical and Procedural Characteristics

	Positive (N = 32)	Indeterminate (N = 24)	Negative (N = 80)
**Clinical Characteristics**			
Age (Years ± S.D.)	63.0 ± 8.5	55.1 ± 9.1	56.0 ± 11.0
Male (%)	75.0	87.5	88.6
% LVEF (Mean ± S.D.)	51 ± 12	54 ± 10	55 ± 11
Pre-PCI 3 vessel CAD (%)	18.8	4.2	6.6
Pre-PCI CCS Class III-IV (%)	65.6	29.2	52.5
Current Smoker (%)	50.0	66.7	63.3
Hyperlipidemia (%)	68.8	77.3	67.9
Diabetes Mellitus (%)	12.5	25.0	20.5
Hypertension (%)	62.5	62.5	48.7
**Procedural Characteristics**			
Single-vessel PCI (%)	81.3	100	88.8
Stent inserted (%)	87.5	75.0	86.3
Lesion location (%)			
Left anterior descending	50.0	58.3	40.0
Left circumflex	18.7	20.8	17.5
Right coronary	31.3	20.8	42.5
ACC/AHA lesion class B2/C* (%)	61.3	73.9	45.7
Post-PCI TIMI-3 flow (%)*	90.6	100	98.7
% Diameter Stenoses (mean ± S.D.)			
% Pre-PCI	86 ± 13	86 ± 12	88 ± 12
% Post-PCI	7 ± 13	5 ± 7	4 ± 10

SD – Standard Deviation; CCS – Canadian Cardiovascular Society; MI – Myocardial Infarction; CABG – Coronary artery Bypass Graft Surgery; PCI – Percutaneous Coronary Intervention; LVEF – Left Ventricular Ejection Fraction; CAD – Coronary Artery Disease; TIMI – Thrombolysis In Myocardial Infarction; ACC/AHA – American College of Cardiology/American Heart Association; PCI – Percutaneous Coronary Intervention

* If >1 lesion underwent PCI, refers to the first lesion that was treated.

**Table 2:** Six-week ETT results

	Positive ETT (N = 32)	Indeterminate ETT (N = 24)	Negative ETT (N = 80)
Weeks after index PCI (Mean ± S.D.)	6.6 ± 2.9	7.1 ± 2.5	6.4 ± 1.8
% Patients on anti-anginals*	90.3	95.8	82.3
Beta-blockers	80.6	87.5	77.2
Calcium-channel blockers	22.5	33.3	23.1
Long-acting nitrates	25.0	25.0	23.1
Baseline ECG (%)			
Normal	54.8	50.0	59.1
Non-specific ST-T abnormalities	12.9	12.5	7.6
Previous MI†	25.8	29.2	22.7
LBBB	3.2	4.2	0.0
RBBB	0.0	4.2	6.1
LVH	3.2	0.0	3.0
Adequate for diagnosis of ischemia‡	87.1	83.3	95.5
% MPHR achieved (Mean ± S.D.)	88.0 ± 17.4	91.1 ± 26.6	92.0 ± 18.0
% achieving ≥ 85% MPHR	65.6	50.0	72.5
Number of METS achieved (Mean ± S.D.)	8.4 ± 2.5	9.5 ± 3.6	9.5 ± 2.9
Rate-Pressure Product (Mean ± S.D.)	23,936 ± 5,934	23,683 ± 5,612	25,247 ± 6,623
Duke Treadmill Score	0.7 ± 5.0	5.5 ± 4.3	7.8 ± 3.7
Development of exertional angina:			
Yes	56.3	0.0	0.0
No	37.5	66.7	100
Indeterminate	6.2	33.3	0.0
Reversible ischemia by ECG:			
Yes	87.5	0.0	0.0
No	9.4	12.5	100
Indeterminate	3.1	87.5	0.0

SD – Standard Deviation; METS – Metabolic Equivalents; PCI – Percutaneous Coronary Intervention MPHR – Maximum Predicted Heart Rate; MI – Myocardial Infarction; LVH – Left Ventricular Hypertrophy; LBBB – Left Bundle Branch Block; RBBB – Right Bundle Branch Block

* Patients prescribed calcium channel blockers, beta blockers or long-acting nitrates on discharge following index PCI.

† Either Q-wave or Non-Q wave

‡ Patients with ST-depression <1.0 mm on the resting ECG without LVH or LBBB

**Table 3:** Clinical Events and Cardiac Procedures at 9-months

	Positive ETT (N = 32)	Indeterminate ETT (N = 24)	Negative ETT (N = 80)	P-value*
Death N (%)	0 (0.0)	1 (4.2)	1 (1.3)	>0.99
Myocardial Infarction N (%)	0 (0.0)	1 (4.2)	2 (2.5)	>0.99
Unstable Angina N (%)	7 (21.9)	3 (13.0)	9 (11.3)	0.23
Composite Clinical Events N (%)	7 (21.9)	5 (20.8)	12 (15.0)	0.25
				
Repeat Angiography N(%)	6 (18.8)	3 (12.5)	5 (6.3)	0.07
Repeat PCI N (%)	4 (12.5)	2 (8.3)	2 (2.6)	0.06
CABG N (%)	1 (3.1)	1 (4.2)	0 (0.0)	0.29
Composite Cardiac Procedures N(%)	11 (34.4)	6 (25.0)	7 (8.8)	≤ 0.01

PCI – Percutaneous Coronary Intervention

CABG – Coronary Artery Bypass Graft Surgery

* P-value for Positive vs. Negative ETT results

**Table 4:** Univariate and multivariate regression analyses – variables predictive of clinical events

	Odds Ratio	95% CI	P-value
**Univariate Analysis**			
Diabetes	3.25	1.18–8.96	0.02
PCI of LCx	2.49	0.89–6.96	0.08
% Residual Stenosis	1.07	1.02–1.11	0.002
Duke Treadmill Score	0.92	0.84–1.01	0.077
METS achieved	0.85	0.71–1.01	0.057
Stenting	0.19	0.07–0.52	0.001
≥ 85% MPHR achieved	0.17	0.06–0.45	0.0004
Positive ETT	1.66	0.61–4.5	0.32
Negative ETT	0.52	0.21–1.32	0.16
			
**Multivariate Analysis**			
Stenting	0.15	0.047–0.479	0.0014
≥ 85% MPHR achieved	0.14	0.048–0.417	0.0004

CI – Confidence Interval

LCx – Left Circumflex

MPHR – Maximum Predicted Heart Rate
